# Mining novel biomarkers for prognosis of gastric cancer with serum proteomics

**DOI:** 10.1186/1756-9966-28-126

**Published:** 2009-09-09

**Authors:** Fu-Ming Qiu, Jie-Kai Yu, Yi-Ding Chen, Qi-Feng Jin, Mei-Hua Sui, Jian Huang

**Affiliations:** 1 Department of Oncology, 2nd Affiliated Hospital, Zhejiang University School of Medicine, 88 Jiefang Rd, Hangzhou, PR China; 2 Cancer Institute of Zhejiang University, 88 Jiefang Rd, Hangzhou, PR China; 3 Department of Pathology and Laboratory Medicine, Medical University of South Carolina, Charleston, SC 29425, USA

## Abstract

**Background:**

Although gastric caner (GC) remains the second cause of cancer-related death, useful biomarkers for prognosis are still unavailable. We present here the attempt of mining novel biomarkers for GC prognosis by using serum proteomics.

**Methods:**

Sera from 43 GC patients and 41 controls with gastritis as Group 1 and 11 GC patients as Group 2 was successively detected by Surface Enhanced Laser Desorption/ionization Time of Flight Mass Spectrometry (SELDI-TOF-MS) with Q10 chip. Peaks were acquired by Ciphergen ProteinChip Software 3.2.0 and analyzed by Zhejiang University-ProteinChip Data Analysis System (ZJU-PDAS). CEA level were evaluated by chemiluminescence immunoassay.

**Results:**

After median follow-up periods of 33 months, Group 1 with 4 GC patients lost was divided into 20 good-prognosis GC patients (overall survival more than 24 months) and 19 poor-prognosis GC patients (no more than 24 months). The established prognosis pattern consisted of 5 novel prognosis biomarkers with 84.2% sensitivity and 85.0% specificity, which were significantly higher than those of carcinoembryonic antigen (CEA) and TNM stage. We also tested prognosis pattern blindly in Group 2 with 66.7% sensitivity and 80.0% specificity. Moreover, we found that 4474-Da peak elevated significantly in GC and was associated with advanced stage (III+IV) and short survival (*p *< 0.03).

**Conclusion:**

We have identified a number of novel biomarkers for prognosis prediction of GC by using SELDI-TOF-MS combined with sophisticated bioinformatics. Particularly, elevated expression of 4474-Da peak showed very promising to be developed into a novel biomarker associated with biologically aggressive features of GC.

## Background

Gastric cancer (GC) is the second leading cause of cancer-related death in the world and remains the top killing cancer in Asia including China [1,2]. Though GC mortality has decreased markedly in most areas of the world, it is an aggressive malignancy and is still difficult to be detected at early stage [3]. Early GC (EGC) tends to be detected in countries with mass screening regimen using endoscopy and radiography. However, the perceived inconvenience, and discomforts caused by endoscopy and radiation have resulted in low compliance. The majority of GC patients are diagnosed at an advanced stage and died in 24 months after operation because of recurrence and metastasis, with only 27% 5-year overall survival rate in patients with extended local resection [4]. Thus, it is of clinical importance to identify GC patients with poor prognosis for intense treatment.

TNM staging system is used world-widely to direct therapeutic decision, predict prognosis, and stratify patients into distinct groups with different risks for tumor-related death [5]. However, due to intrinsic heterogeneity, cancer patients with equivalent TNM stage, type and grade may have quite different response to treatment and clinical behavior. Moreover, changes of currently used serum-derived biomarkers of GC such as carcinoembryonic antigen (CEA), CA 19-9 and CA 72-4 usually appear in advanced stage, and therefore have limited value in clinics for predicting prognosis (lower than 40%) [6,7]. Although the combined use of these biomarkers have shown certain improvement, their value is still far from ideal [8-10].

Progresses in proteomics have presented new horizon and led to novel techniques for mining serum biomarkers for the detection of various carcinomas including GC [11]. SELDI-TOF-MS coupled with sophisticated bioinformatics offers a sensitive, high-throughput, and rapid approach for analyzing complex mixture of protein and peptide [12,13]. Moreover, it is capable of inspecting the whole proteome of serum and this meets our needs for mining biomarkers based on disease condition. This approach has been used to establish detection patterns for various tumors [14], but its value in mining biomarkers for prediction of prognosis and stage has seldom been evaluated.

In the present prospective study, we classified GC patients into good-prognosis group and poor-prognosis group based on its survival characteristics. We discovered 5 novel biomarkers related to prognosis of GC by establishing prognosis pattern with biomarker discovery set and validated in an independent set. More importantly, we found that peak at 4474 Da was significantly elevated in poor-prognosis GC patients and patients with advanced TNM stage.

## Methods

### Patient demographics

This study was approved by institutional review board and conducted under the informed consent of patients. Forty three consecutive GC patients and 41 gastritis patients with dyspeptic symptoms as Group 1 in 2^nd ^affiliated hospital of Zhejiang University School of Medicine, China, from February 2003 and October 2004 were initially enrolled for biomarker mining in this study. All of the 43 GC patients underwent surgical operations, including 39 curative resections with D2 lymphadenectomy and 4 palliative operations due to the presence of metastasis. All participants were histologically verified adenocarcinoma or gastritis by gastroscopy. Median age of GC patients was 58 years (range, 36~76 years) and that of controls was 51 years (range, 38~73 years) (T-test *p *= 0.09). Sex distribution was similar between GC patients (29 males and 14 females) and controls (28 males and 13 females) (T-test *p *= 0.93). Clinical stage was assessed according to AJCC TNM stage (6^th ^edition 2002).

**Figure 1:**
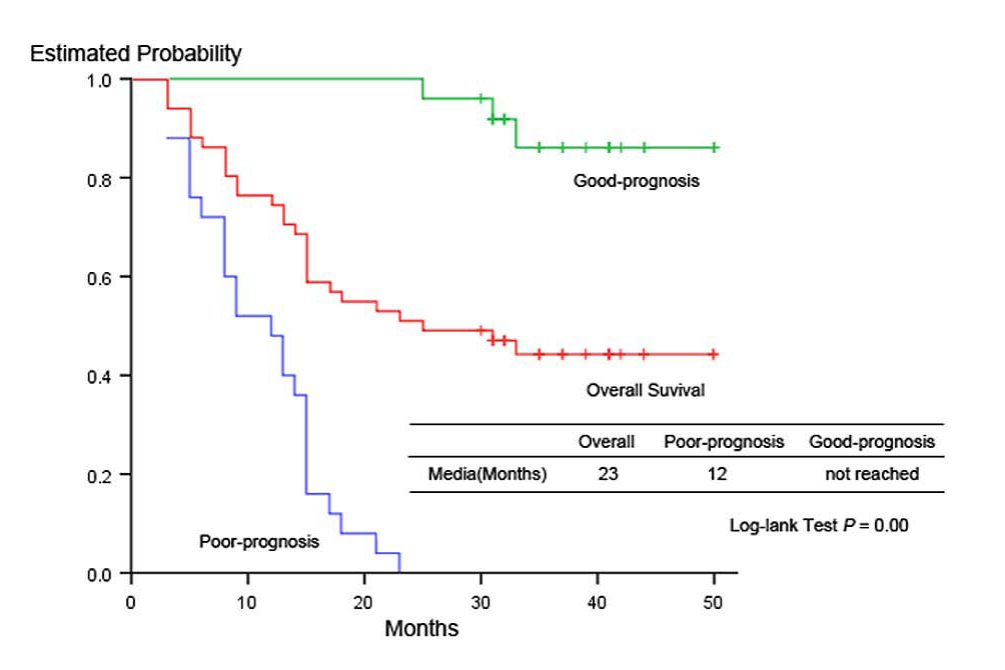
**Survival curve for all included GC patients, good-prognosis and poor-prognosis GC patients**. The media survival time (months) for all included GC patients (n = 54), poor- prognosis (n = 25) and good-prognosis GC patients (n = 25) was 23, 12 and not reached, respectively. There was significantly statistical difference between poor-prognosis and good-prognosis groups (Log-rank test *p *= 0.00).

Eleven GC patients with curative resection were subsequently enrolled as Group 2 for blind test. Post-operative follow-up visits were performed every 3 months for the first 2 years and then every 6 months up to 63 months or death. With 1 GC patient from Group 1 died of surgical complication, the follow-up rate was 94.3% (50/53) and all 3 lost patients were also in Group 1. For the remaining 50 GC patients, median postoperative follow-up periods were 33 months (3 to 63 months). Based on the fact that median survival of GC is 24 months, we defined GC patients with overall survival (OS) no more than 24 months as poor-prognosis group, and others as good-prognosis [15,16]. As presented in Fig. 1, the media survival time (months) for all included GC patients (n = 54), poor- prognosis (n = 25) and good-prognosis GC patients (n = 25) was 23, 12 and not reached, respectively. We thus defined 20 patients as good-prognosis and left 19 as poor-prognosis GC patients in Group 1, and 5 patients as good-prognosis and left 6 as poor-prognosis in Group 2. None of the gastritis patients developed GC during the period and after follow-up for 48 months.

### Blood processing and peak detection

All blood specimens were collected in the fasted state in the morning before initiation of any treatment. Every sample was rest at room temperature for 1-2 hours, centrifuged at 3 × *g *for 10 minutes. Serum samples were then aliquoted into eppendorf tubes and frozen at -80°C until use. Group 1 and 2 were detected in a separated date according the following methods.

Serum samples were thawed on ice and centrifugated at 10 × *g *for 4 minutes with supernatants retained before detection. Ten μL of U9 denaturing buffer (9 M Urea, 2% CHAPS, 1% DTT) was added to 5 μL of each serum sample in a 96-well cell culture plate and agitated on a platform shaker for 30 minutes at 4°C. The U9/serum mixture was then loaded to 185 μL binding buffer (50 mM Tris-HCl, pH9) and agitated again for 2 minutes at 4°C. Meanwhile, Q10 chips were placed in the Bioprocessor (Ciphergen Biosystems) and pre-activated with binding buffer (200 μL) for 5 minutes twice. The diluted samples (100 μL) were then pipetted onto the spots on ProteinChip array. After incubation for 60 minutes at 4°C, the chips were washed three times with binding buffer (3 × 200 μL) and twice with deionized water (2 × 200 μL). Finally, the chips were removed from the bioprocessor and air-dried. Before SELDI-TOF-MS analysis, saturated energy-absorbing molecule solution (sinapinic acid in 50% ACN and 0.5% TFA, 2 × 0.5 μL) was applied to each spot twice and air-dried.

The chips were detected on the PBS-II plus mass spectrometer reader (Ciphergen Biosystems) and peak detection was performed using the Ciphergen ProteinChip Software 3.2.0. Calibration of mass accuracy was determined using the all-in-one peptide molecular mass standard. Data were collected by averaging 140 laser shots with intensity of 170 and detector sensitivity of 8. The highest mass of 60,000 *m/z *and optimized range of 2,000-20,000 Da were set for analysis.

### Serum CEA measurement

CEA level of all serum samples were evaluated in parallel with SELDI-TOFMS analysis by chemiluminescence immunoassay (CEA Regent Kit, Abbott Diagnostics). Assays were carried out according to the manufacturer's instructions by using ARCHITECT i2000 SR. The cutoff value of CEA for prognosis prediction, detection and stage discrimination of GC was set at 5 ng/mL.

### Bioinformatics and statistic analysis

Bioinformatics and biostatistics were operated by Zhejiang University-Proteinchip Data Analysis System (ZJU-PDAS, ), which was designed by Yu and based on MATLAB Web Server 1.2.4 (The MathWorks Inc.). ZJU-PDAS and detailed protocols have been described in our previous report [17]. Spectra were denoised by undecimated discrete wavelet transform, based on the version 2.4 of the Rice Wavelet Toolbox, followed by subtraction of baseline and calibration of mass. The detected peaks were filtered by S/N more than 3 and combined peaks in relative mass by 0.3%. Peaks appeared in more than 10% of spectra were defined as peaks cluster. Then we constructed a non-linear supportive vector machine (SVM) classifier with a radial based function kernel to discriminate the different groups. Leave-one-out cross-validation approach was applied to estimate the accuracy of the classifier. This approach leaves one sample out to be test set and the remaining samples as the training set. The process continues until each sample has been held in reserve one time as a test sample. Power of each peak in discriminating different groups was evaluated by the *p *value of Wilcoxon Rank Sum test. The top 10 peaks with the least *p *value were selected and randomly input into SVM in combination. The SVM model which achieved the highest Youden's Index was determined as the final pattern and the peaks were selected as candidate biomarkers. Receiver operating curve (ROC) and survival curve was performed with SPSS package version 11.0.

## Results

### Assay reproducibility

The reproducibility of the proteomic approach was determined by repeating one sera mixture 11 times using standard procedures described above. The average coefficient of variance (CV) for the selected peaks with normalized intensity was 17.2% and the CV for selected peak mass was 0.03%.

### Biomarkers for prognosis prediction and blind test

**Figure 2:**
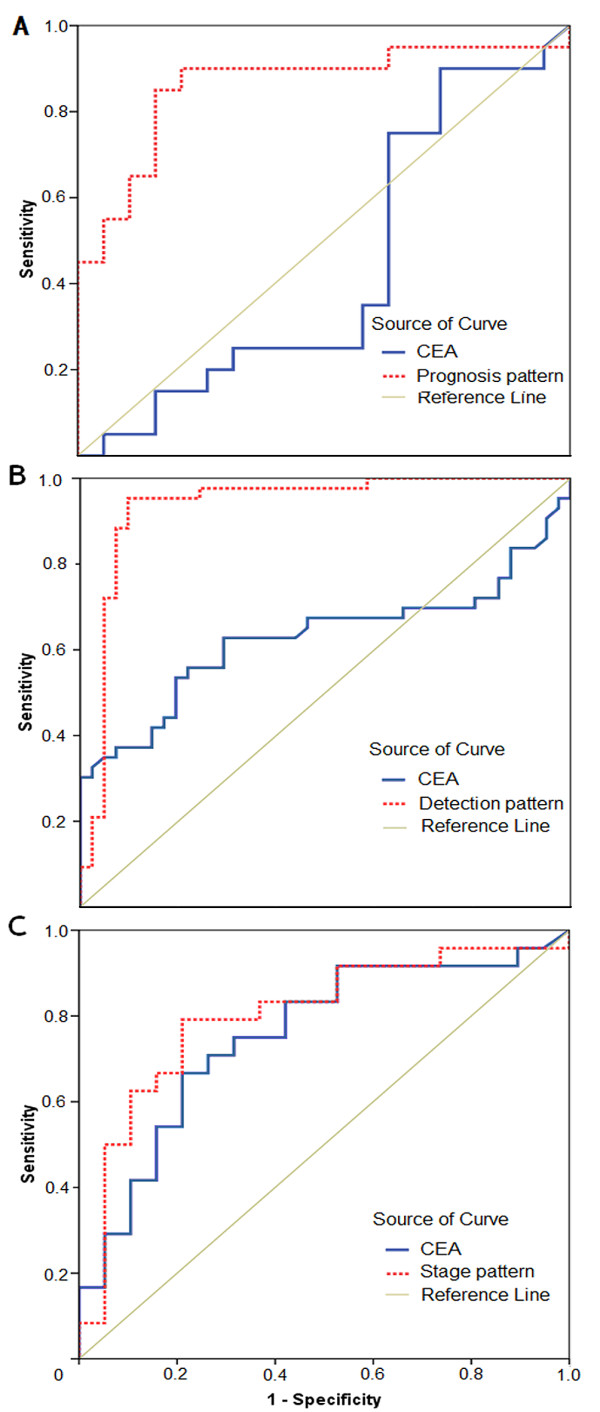
**The areas under Receiver Operating Characteristic (ROC) curves for prognosis pattern and CEA (A), detection pattern and CEA (B), stage pattern and CEA (C)**.

**Figure 3:**
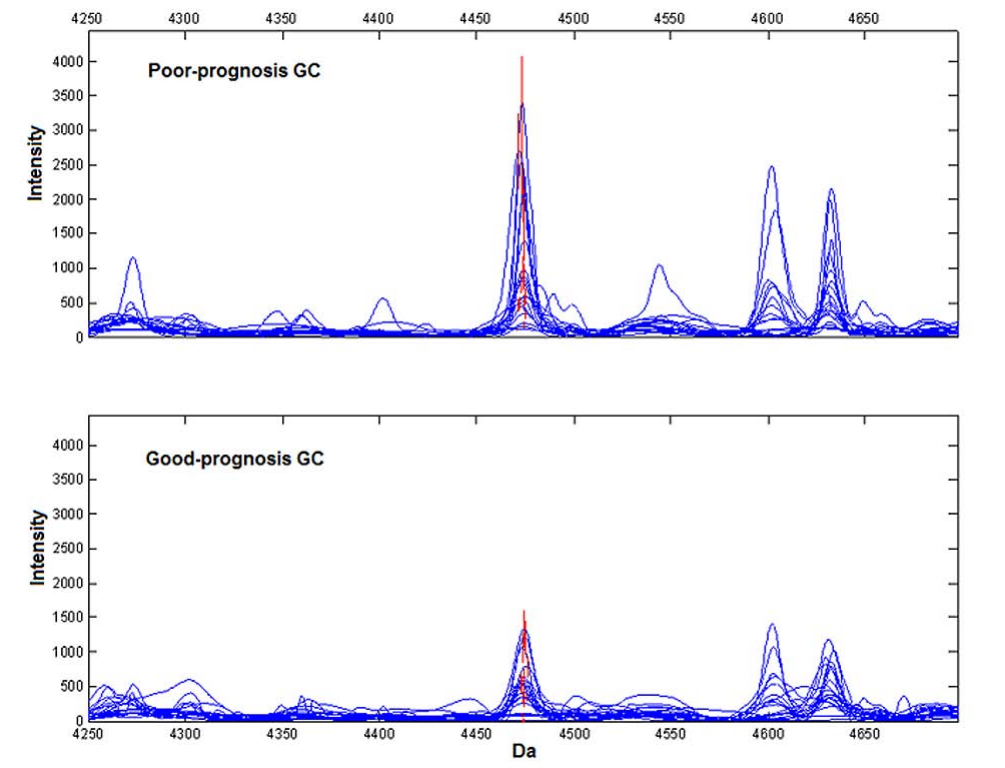
**Representative expression of the peak at 4474 Da (red) in prognosis pattern**. Peak at 4474 Da was significantly higher in poor-prognosis GC (upper panel), compared with good-prognosis GC (lower panel) in biomarker mining set. Wilcoxon Rank Sum *p *= 0.04.

Total 50 peaks were qualified for establishing prognosis pattern by comparing proteomic spectrum of 20 good-prognosis GC patients with 19 poor-prognosis GC patients in Group 1. The established prognosis pattern consisted of 5 prognosis biomarkers with peaks at 4474, 4542, 6443, 4988, 6685 Da (see Additional file 1). This prognosis pattern distinguished poor-prognosis group from good-prognosis with sensitivity of 84.2% (16/19) and specificity of 85.0% (17/20), while the sensitivity and specificity of CEA only reached 52.6 (10/19) and 70.0 (14/20) correspondingly (Table 1). Moreover, the area under ROC curve for the pattern was 0.861 (95% CI, 0.735 to 0.986), significantly higher than 0.436 (95% CI, 0.246 to 0.625) for CEA (Fig 2A). Peak at 4474 Da was found to be the most informative biomarker with the area under ROC curve of 0.695 (95% CI, 0.527 to 0.862), and with significantly higher expression level in poor-prognosis group (Wilcoxon Rank Sum *p *= 0.04, Fig 3). Considering that TNM staging system has been commonly used to predict prognosis, we also evaluated the predictive ability of TNM stage by defining patients with stage I+II (n = 18) as good prognosis and stage III+IV (n = 21) as poor prognosis. However, our data indicate that the sensitivity and specificity of TNM stage for predicting GC patients with poor prognosis were 66.7% (14/21) and 72.2% (13/18) respectively, both of which were inferior compared to the prognosis pattern established in our study.

**Table 1:** Descriptive Statistics of Prognosis, Detection and Stage patterns for GC compared with CEA correspondingly.

**Biomarkers**	**ROC**	**Sensitivity (%)**	**Specificity (%) **
Prognosis pattern	0.861	84.2 (16/19)	85.0 (17/20)
CEA	0.436	52.6 (10/19)	70.0 (14/20)
Detection pattern	0.934	95.4 (41/43)	90.2 (37/41)
CEA	0.628	34.9 (15/43)	95.1 (39/41)
Stage pattern	0.800	79.2 (19/24)	78.9 (15/19)
CEA	0.753	50.0 (12/24)	84.2 (16/19)

**Figure 4:**
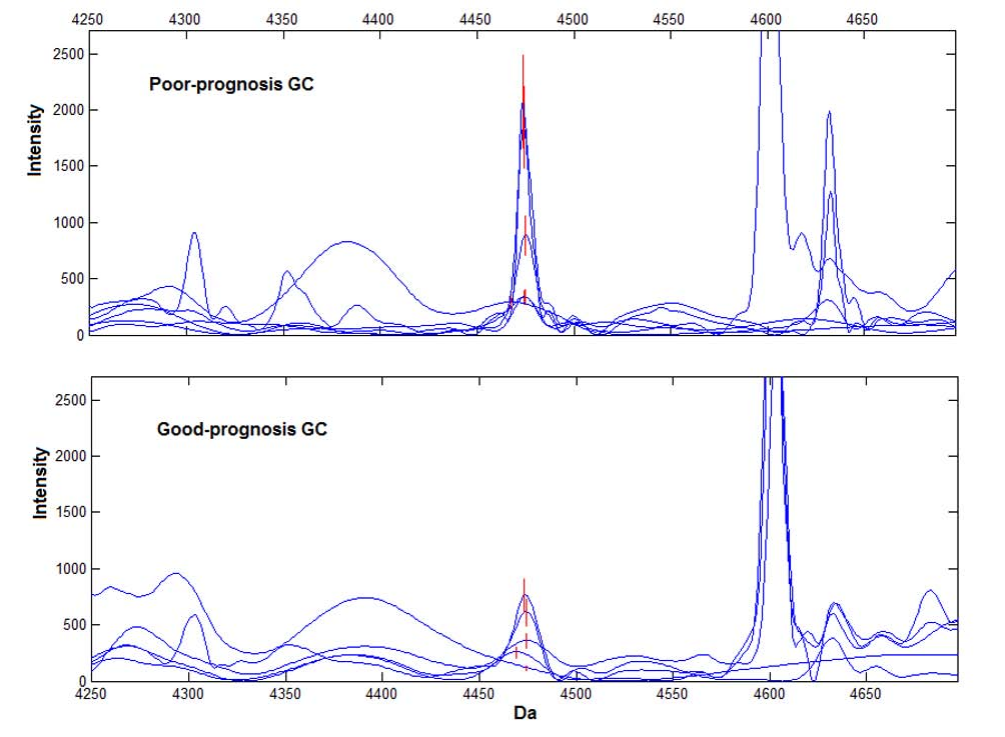
**Representative expression of the peak at 4474 Da (red) in blind test set for prognosis pattern**. Peak at 4474 Da was high expressed in poor-prognosis GC (upper panel), compared with good-prognosis GC (lower panel) in blind test with 5 good-prognosis and 6 poor-prognosis GC patients.

Group 2 with 5 good-prognosis and 6 poor-prognosis GC patients were analyzed to blind test the prognosis prediction pattern. The pattern acquired 66.7% (4/6) sensitivity and 80.0% (4/5) specificity, and peak at 4474 Da had significantly higher expression level in poor-prognosis GC patients than good-prognosis patients (Intensity 965.42 ± 809.28 *versus *425.31 ± 263.19, Fig 4).

### Roles of prognosis biomarkers in GC pathogenesis

**Figure 5:**
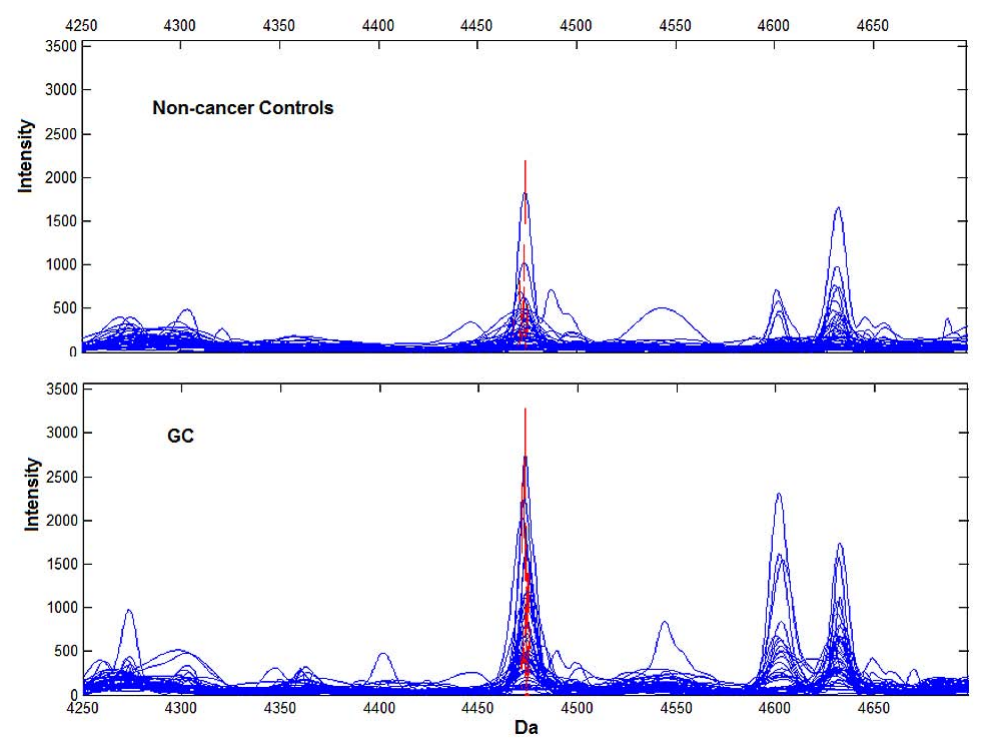
**Representative expression of the peak at 4474 Da (red) in detection pattern**. Peak at 4474 Da was significantly higher in GC (lower panel), compared with non-cancer controls (upper panel). Wilcoxon Rank Sum *p *< 0.001.

To investigate the role of prognosis biomarkers in carcinogenesis of GC, we compared the proteomic spectrum of 43 GC patients with 41 non-cancer controls in Group 1 and total of 34 qualified peaks were determined. Six peaks at 3957, 4474, 4158, 8938, 3941 and 4988 Da, respectively, were identified as potential biomarkers for carcinogenesis of GC and therefore composed the detection pattern (see Additional file 1). Sensitivity and specificity for our established detection pattern were 95.4% (41/43) and 90.2% (37/41) respectively, while the parallel analysis of serum CEA only achieved 34.9% (15/43) and 95.1% (39/41), respectively (Table 1). The areas under ROC curve was 0.934 (95% CI, 0.872 to 0.997) for the detection pattern and 0.628 (95% CI, 0.503 to 0.754) for CEA (Fig 2B). Though peak at 3957 Da was the most useful biomarker for screening, it highly expressed in non-cancer controls. Among biomarkers up-regulated in GC, peak at 4474 Da was the most powerful discriminative biomarker with ROC 0.716 (95% CI, 0.605 to 0.826; Wilcoxon Rank Sum *p *< 0.001) (Fig. 5).

**Figure 6:**
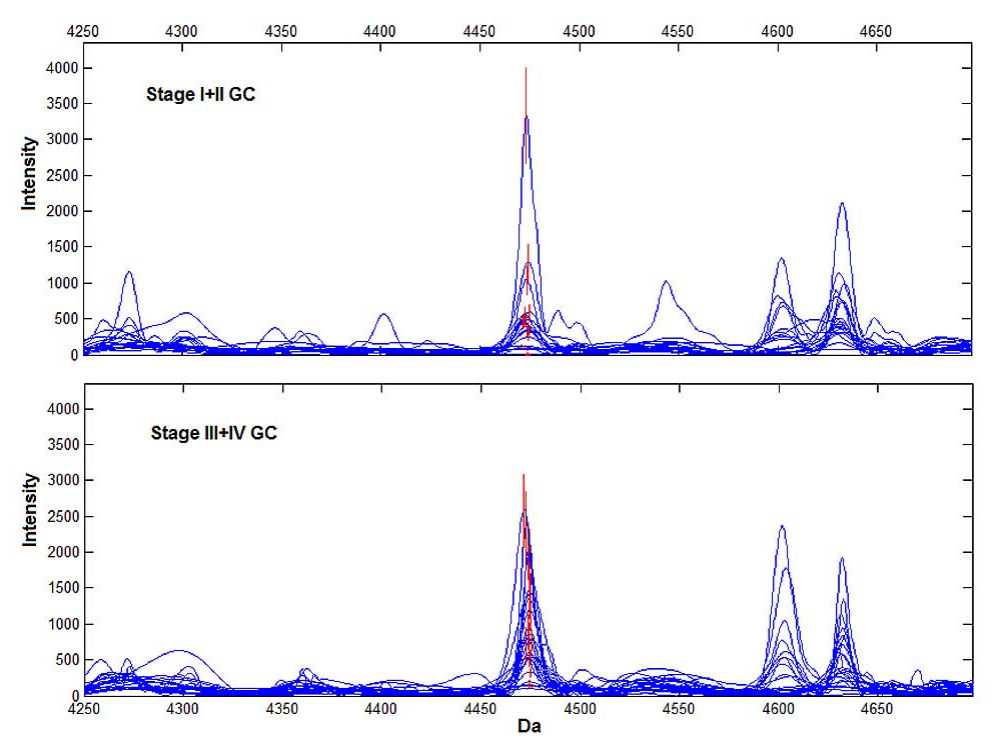
**Representative expression of the peak at 4474 Da (red) in stage pattern**. Peak at 4474 Da was significantly higher in stage III+IV GC (lower panel), compared with stage I/II GC (upper panel). Wilcoxon Rank Sum *p *= 0.01.

To explore if the prognosis biomarkers also play a role in GC progression, 19 patients with stage I+II and 24 with stage III+IV from Group 1 were analyzed for stage discrimination. Overall, 36 peaks were qualified and finally 6 peaks at 4474, 4060, 3957, 9446, 4988 and 5075 Da, respectively, constructed the stage discriminating pattern (see Additional file 1). This pattern could discriminate stage III+IV with 79.2% (19/24) sensitivity and 78.9% (15/19) specificity, while CEA only achieved 50.0% (12/24) and 84.2% (16/19), respectively (Table 1). The area under ROC curve was 0.800 (95% CI, 0.661 to 0.939) for the established pattern and 0.753 (95% CI 0.60~0.90) for CEA (Fig 2C). Interestingly, peak at 4474 Da was also the most powerful biomarker for GC stage discrimination with ROC of 0.732 (95% CI, 0.576 to 0.889, Wilcoxon Rank Sum *p *= 0.01) and with significantly higher expression level in stage III+IV (Fig 6).

## Discussion

GC is a heterogeneous disease and survival benefits could be gained through early detection and intensive post-operative treatment for selected patients. Evidence from large randomized controlled trails supported TNM stage is the most important index for postoperative treatment. Yet inferior survival benefit made the majority of patients over treated and we urgently need robust prognostic biomarker to alter this fatal outcome. Unfortunately, despite efforts with pharmacogemomics or gene-expression data, biomarkers with high and reliable predictive value for GC prognosis are still unavailable. Intrinsic genetic heterogeneity of GC have supported that panels of multiple biomarkers may improve the predictive efficiency. Serum proteomics conducted by SELDI-ProteinChip platform with bioinformatics to associate complex patterns with disease has been attractive, as it is easily accessible, non-invasive and clinically applicable. Novel biomarkers detected by such approach have been reported in various tumors, including prostate cancer [18,19], ovarian cancer [20,21], brain cancer [22], colorectal cancer [23,24], breast cancer [25,26], lung cancer [27] and GC [28]. This approach has yielded informative biomarker profiles in cancer detection with higher sensitivity and specificity, but none of these studies have investigated the correlation between serum protein profiles with prognosis of GC [29].

Though many efforts have been devoted to improve early detection of GC, the majority of patients were diagnosed at advanced stage. Identification of patients with potential poor-prognosis would help us to optimize the clinical treatment for GC patients. Histological grade, anatomically based TNM staging system, serum biomarkers, genes and other factors have been used to predict prognosis so far [5,15,30,31]. Currently, TNM staging system remains the most widely used prognostic model, while newly emerging biomarkers such as CEA, CA72-4 or its combination may provide additional prognostic information. For example, Kochi et al demonstrated that patients with elevated serum CEA levels were at significantly higher risk of having GC recurrence than those with normal levels [8]. However, as shown in several studies including the present study, these serum biomarkers have limited predictive value due to their low sensitivities [6-9]. Therefore, seeking new biomarkers with higher and more reliable predictive value for malignancies has been of great interest in both research and clinical settings.

After median follow-up period of 33 months, we divided 50 patients with follow-up result into biomarker mining set (Group 1) and independent blind test set (Group 2). Our data indicated that the prognosis pattern consisted of 5 potential prognosis biomarkers (peaks at 4474, 4542, 6643, 4988 and 6685 Da) could distinguish the two different groups with 85.0% sensitivity and 84.2% specificity, both of which are significantly higher than traditional TNM stage and/or serum CEA. More importantly, we discovered that 4474-Da peak, a novel peak has not been reported previously, was the most informative peak for prognosis prediction. To further confirm these findings, a blind test with 11 independent GC patients was performed. Our data showed that the sensitivity and specificity of the prognosis pattern were 66.7% and 80.0%, respectively. Moreover, a significantly higher expression level of peak at 4474 Da in poor-prognosis GC group was also observed in independent blind test set.

Additionally, we investigated the role of prognosis biomarkers in the carcinogenesis and progression of GC. With comparison of GC and gastritis group, we confirmed that prognosis biomarkers with peak at 4474, 4988 Da were highly expressed in GC group and indicated that they may play a role in carcinogenesis of GC. Furthermore, peak at 4474 Da may contribute to the occurrence of GC owing to its most significantly elevated expression in GC. With comparison of different stage of GC, we discovered that 4474-Da peak especially up-regulated in GC with advanced stage. In a word, peak at 4474 Da was not only a candidate biomarker for prognosis prediction, but also a biomarker play an important role in the carcinogenesis and development of GC.

## Conclusion

In this study, by using SELDI-TOF-MS combined with sophisticated bioinformatics, we have identified a number of novel biomarkers for prognosis prediction of GC. Moreover, peak at 4474 Da was found to be significantly associated with aggressive characteristics of GC. Thus, identification of peaks at 4474Da may facilitate prognosis prediction and decision making for clinically intensive treatment and worthy of further investigation on a larger scale. In the coming era of personalized medicine, protein profiling attempts like this study may provide important basis for individualized therapy to cancer patients.

## Competing interests

The authors declare that they have no competing interests.

## Authors' contributions

JH designed this study. FMQ and JQF collected samples and followed up patients. FMQ and YDC finished SELDI-TOF-MS detection and CEA measurement. JKY finished bioinformatics and statistic analysis. FMQ, MHS and JH drafted the manuscript. All authors read and approved the final manuscript.

## Supplementary Material

Additional file 1 **Descriptive Statistics of peaks in three patterns for GC**. The data provided list *p *value, ROC and intensity of all peaks in prognosis, detection and stage patterns in GC. Click here for file

## References

[B1] Parkin DM, Bray F, Ferlay J, Pisani P (2005). Global cancer statistics, 2002. CA Cancer J Clin.

[B2] Yang L (2006). Incidence and mortality of gastric cancer in China. World J Gastroenterol.

[B3] Jemal A, Thomas A, Murray T, Thun M (2002). Cancer statistics, 2002. CA Cancer J Clin.

[B4] Martin RC, Jaques DP, Brennan MF, Karpeh M (2002). Extended local resection for advanced gastric cancer: increased survival versus increased morbidity. Ann Surg.

[B5] Klein Kranenbarg E, Hermans J, van Krieken JH, Velde CJ van de (2001). Evaluation of the 5th edition of the TNM classification for gastric cancer: improved prognostic value. Br J Cancer.

[B6] Kodera Y, Yamamura Y, Torii A, Uesaka K, Hirai T, Yasui K, Morimoto T, Kato T, Kito T (1996). The prognostic value of preoperative serum levels of CEA and CA19-9 in patients with gastric cancer. Am J Gastroenterol.

[B7] Marrelli D, Roviello F, De Stefano A, Farnetani M, Garosi L, Messano A, Pinto E (1999). Prognostic significance of CEA, CA 19-9 and CA 72-4 preoperative serum levels in gastric carcinoma. Oncology.

[B8] Kochi M, Fujii M, Kanamori N, Kaiga T, Kawakami T, Aizaki K, Kasahara M, Mochizuki F, Kasakura Y, Yamagata M (2000). Evaluation of serum CEA and CA19-9 levels as prognostic factors in patients with gastric cancer. Gastric Cancer.

[B9] Aloe S, D'Alessandro R, Spila A, Ferroni P, Basili S, Palmirotta R, Carlini M, Graziano F, Mancini R, Mariotti S, Cosimelli M, Roselli M, Guadagni F (2003). Prognostic value of serum and tumor tissue CA 72-4 content in gastric cancer. Int J Biol Marker.

[B10] Ucar E, Semerci E, Ustun H, Yetim T, Huzmeli C, Gullu M (2008). Prognostic value of preoperative CEA, CA 19-9, CA 72-4, and AFP levels in gastric cancer. Adv Ther.

[B11] Simpson RJ, Bernhard OK, Greening DW, Moritz RL (2008). Proteomics-driven cancer biomarker discovery: looking to the future. Curr Opin Chem Biol.

[B12] Petricoin EF, Liotta LA (2004). SELDI-TOF-based serum proteomic pattern diagnostics for early detection of cancer. Curr Opin Biotechnol.

[B13] Wright GL (2002). SELDI proteinchip MS: a platform for biomarker discovery and cancer diagnosis. Expert Rev Mol Diagn.

[B14] Ludwig JA, Weinstein JN (2005). Biomarkers in cancer staging, prognosis and treatment selection. Nat Rev Cancer.

[B15] Dicken BJ, Bigam DL, Cass C, Mackey JR, Joy AA, Hamilton SM (2005). Gastric adenocarcinoma: review and considerations for future directions. Ann Surg.

[B16] Hohenberger P, Gretschel S (2003). Gastric cancer. Lancet.

[B17] Wang JX, Yu JK, Wang L, Liu QL, Zhang J, Zheng S (2006). Application of serum protein fingerprint in diagnosis of papillary thyroid carcinoma. Proteomics.

[B18] Adam BL, Qu Y, Davis JW, Ward MD, Clements MA, Cazares LH, Semmes OJ, Schellhammer PF, Yasui Y, Feng Z, Wright GL (2002). Serum protein fingerprinting coupled with a pattern-matching algorithm distinguishes prostate cancer from benign prostate hyperplasia and healthy men. Cancer Res.

[B19] Qu Y, Adam BL, Yasui Y, Ward MD, Cazares LH, Schellhammer PF, Feng Z, Semmes OJ, Wright GL (2002). Boosted decision tree analysis of surface-enhanced laser desorption/ionization mass spectral serum profiles discriminates prostate cancer from noncancer patients. Clin Chem.

[B20] Zhang Z, Bast RC, Yu Y, Li J, Sokoll LJ, Rai AJ, Rosenzweig JM, Cameron B, Wang YY, Meng XY, Berchuck A, Van Haaften-Day C, Hacker NF, de Bruijn HW, Zee AG van der, Jacobs IJ, Fung ET, Chan DW (2004). Three biomarkers identified from serum proteomic analysis for the detection of early stage ovarian cancer. Cancer Res.

[B21] Yu JK, Zheng S, Tang Y, Li L (2005). An integrated approach utilizing proteomics and bioinformatics to detect ovarian cancer. J Zhejiang Univ Sci B.

[B22] Liu J, Zheng S, Yu JK, Zhang JM, Chen Z (2005). Serum protein fingerprinting coupled with artificial neural network distinguishes glioma from healthy population or brain benign tumor. J Zhejiang Univ Sci.

[B23] Yu JK, Chen YD, Zheng S (2004). An integrated approach to the detection of colorectal cancer utilizing proteomics and bioinformatics. World J Gastroenterol.

[B24] Chen YD, Zheng S, Yu JK, Hu X (2004). Artificial neural networks analysis of surface-enhanced laser desorption/ionization mass spectra of serum protein pattern distinguishes colorectal cancer from healthy population. Clin Cancer Res.

[B25] Hu Y, Zhang S, Yu J, Liu J, Zheng S (2005). SELDI-TOF-MS: the proteomics and bioinformatics approaches in the diagnosis of breast cancer. Breast.

[B26] Laronga C, Becker S, Watson P, Gregory B, Cazares L, Lynch H, Perry RR, Wright GL, Drake RR, Semmes OJ (2004). SELDI-TOF serum profiling for prognostic and diagnostic classification of breast cancers. Dis Markers.

[B27] Xiao X, Liu D, Tang Y, Guo F, Xia L, Liu J, He D (2004). Development of proteomic patterns for detecting lung cancer. Dis Markers.

[B28] Ebert MP, Meuer J, Wiemer JC, Schulz HU, Reymond MA, Traugott U, Malfertheiner P, Röcken C (2004). Identification of gastric cancer patients by serum protein profiling. J Proteome Res.

[B29] Herrmann K, Walch A, Balluff B, Tänzer M, Höfler H, Krause BJ, Schwaiger M, Friess H, Schmid RM, Ebert MP (2009). Proteomic and metabolic prediction of response to therapy in gastrointestinal cancers. Nat Clin Pract Gastroenterol Hepatol.

[B30] Siewert JR, Bottcher K, Stein HJ, Roder JD (1998). Relevant prognostic factors in gastric cancer: ten-year results of the German Gastric Cancer Study. Ann Surg.

[B31] Hao Y, Yu Y, Wang L, Yan M, Ji J, Qu Y, Zhang J, Liu B, Zhu Z (2008). IPO-38 is identified as a novel serum biomarker of gastric cancer based on clinical proteomics technology. J Proteome Res.

