# Supplementary material for: Mining novel biomarkers for prognosis of gastric cancer with serum proteomics
Source: J Exp Clin Cancer Res. 2009 Sep 9;28(1):126. doi: 10.1186/1756-9966-28-126 (PMC2753349; doi:10.1186/1756-9966-28-126)
Supplement: Additional file 1 — Descriptive Statistics of peaks in three patterns for GC. The data provided list p value, ROC and intensity of all peaks in prognosis, detection and stage patterns in GC. [file 1756-9966-28-126-S1.doc]

**Additional files**

**Additional file 1: Descriptive Statistics of peaks in three patterns for GC.**

**Biomarkers *p* value** **ROC** **Intensity 1 Intensity 2**

**(Mean ± SD)** **(Mean ± SD)**

**Prognosis pattern 0.861 Good-prognosis Poor-prognosis**

4474 0.038907 0.695 516.53 ± 365.19 1115.36 ± 973.37

4542 0.041643 0.692 119.97 ± 83.32 212.46 ± 216.71

6443 0.12569 0.645 1793.32 ± 808.48 2591.38 ± 1646.41

4988 0.15592 0.634 500.63 ± 360.13 356.17 ± 243.72

6685 0.25514 0.608 347.74 ± 137.23 318.42 ± 246.22

**Detection pattern 0.934 Control GC**

3957 1.37E-05 0.776 821.86 ± 408.03 453.39 ± 254.43

4474 0.000673 0.716 300.15 ± 322.32 655.55 ± 620.55

4158 0.003739 0.684 298.53 ± 237.64 399.58 ± 194.36

8938 0.016475 0.652 555.44 ± 431.43 347.01 ± 277.33

3941 0.054361 0.622 494.18 ± 337.49 709.63 ± 481.53

4988 0.069284 0.615 315.23 ± 327.11 361.44 ± 243.56

**Stage pattern** **0.800 Stage I/II Stage III/IV**

4474 0.009878 0.732 572.20 ± 744.92 950.12 ± 711.38

4060 0.089191 0.654 364.79 ± 153.64 293.63 ± 129.19

3957 0.1147 0.643 628.11 ± 303.23 485.04 ± 314.91

9446 0.12044 0.640 608.82 ± 444.38 413.26 ± 379.34

4988 0.12639 0.638 510.16 ± 321.49 373.23 ± 265.95

5075 0.13257 0.636 486.01 ± 336.32 353.75 ± 263.20
